# Urinary Tract Infection Induced Delirium in Elderly Patients: A Systematic Review

**DOI:** 10.7759/cureus.32321

**Published:** 2022-12-08

**Authors:** Chandrani Dutta, Khadija Pasha, Salomi Paul, Muhammad S Abbas, Sondos T Nassar, Tasniem Tasha, Anjali Desai, Anjana Bajgain, Asna Ali, Lubna Mohammed

**Affiliations:** 1 Family Medicine, California Institute of Behavioral Neurosciences & Psychology, Fairfield, USA; 2 Pediatric, California Institute of Behavioral Neurosciences & Psychology, Fairfield, USA; 3 Medicine, California Institute of Behavioral Neurosciences & Psychology, Fairfield, USA; 4 Internal Medicine, California Institute of Behavioral Neurosciences & Psychology, Fairfield, USA; 5 Medicine and Surgery, California Institute of Behavioral Neurosciences & Psychology, Fairfield, USA; 6 Division of Research and Academic Affairs, California Institute of Behavioral Neurosciences & Psychology, Fairfield, USA

**Keywords:** urinary tract infection symptoms in elderly, atypical presentation, infection induced delirium, urine infection, geriatrics, asymptomatic bacteriuria, elderly, delirium, urinary tract infection

## Abstract

Urinary tract infection (UTI) is common in older adults, mainly due to several age-related risk factors. Symptoms of UTI are atypical in the elderly population, like hypotension, tachycardia, urinary incontinence, poor appetite, drowsiness, frequent falls, and delirium. UTI manifests more commonly and specifically for this age group as delirium or confusion in the absence of a fever. This systematic review aims to highlight the relationship between UTI and delirium in the elderly population by understanding the pathologies individually and collectively. A systematic review is conducted by searching PubMed with regular keywords and major Medical Subject Heading (MeSH) keywords, Science Direct, and Google Scholar. The inclusion criteria consisted of studies based on male and female human populations above the age of 65 in the English language, available in full text published between 2017 and 2022. However, the exclusion criteria were animal studies, clinical trials, literature published before 2017, and papers published in any other language except English. A total of 106 articles were identified, and nine final studies were selected after a quality assessment, following which a valid relationship between delirium and UTI was identified in this systematic review.

## Introduction and background

Urinary tract infection (UTI) is a common infection in the elderly, mainly due to age-related risk factors like malnutrition, inadequately controlled diabetes mellitus, poor bladder control leading to urinary retention or incontinence, constipation, long-term hospitalizations, vaginal atrophy, prostate hyperplasia, unhygienic living conditions, and altered mental state [1,2]. UTIs are responsible for around 25% of all geriatric hospitalizations attributing to almost 6.2% of deaths due to infectious diseases and repeated emergency department and office visits yearly [2]. UTI usually presents with localized symptoms like painful urination, new onset or worsening urinary urgency or frequency, and suprapubic pain but symptoms of UTI are atypical in the elderly population. UTI manifests more atypically for this age group as delirium, confusion, dizziness, drowsiness, falls, urinary incontinence, or poor appetite in the absence of fever making the diagnosis of UTI a difficult task as patients are unable to report their urinary symptoms clearly [1-4].

Delirium is often reversible, but patients who develop delirium have prolonged hospital stays and complicated recovery [5]. The etiology of delirium is multifactorial; the most common triggering factor of delirium is found to be an infection in 49.5% of cases (mainly due to UTIs and lung infections) [5]. Early antibiotic treatment for bacteriuria in absence of symptoms of UTI is also one of the precipitating factors of delirium therefore, the management of delirium involving the identification of the triggering factors and focusing on treating them is complicated [6-8]. A significant relationship between UTI and delirium is found [9]; this systematic review explores the relationship in detail. The study aims to direct clinicians towards the most common causes while evaluating a patient with delirium to minimize the delay in diagnosis of underlying etiology and initiation of treatment.

## Review

Methods

As per the guidelines from the Preferred Reporting Items for Systematic Reviews and Meta-Analyses (PRISMA) 2020 statement [10], we conducted this systematic review. The systematic review is conducted by searching PubMed with regular keywords and major Medical Subject Heading (MeSH) keywords, Science Direct, and Google Scholar. Inclusion criteria consisted of studies based on male and female human populations above the age of 65 in the English language, available in full text published between 2017 and 2022. The exclusion criteria were animal studies, clinical trials, literature published before 2017, and articles published in any other language except English.

Thirty-seven literature was found in PubMed, 224 literature in Science Direct, and the first 250 relevant literature out of 12800 were selected from Google Scholar search results. Studies were selected after applying the inclusion/exclusion criteria shown in Table 1.

**Table 1:** Databases, search strategy and keywords, filters, and results found and used in this systematic review

Database name	Search strategy and keywords	Filters applied	Results
PubMed	Urinary tract infection OR ("Urinary Tract Infections/analysis" [Majr] OR "Urinary Tract Infections/diagnosis" [Majr] OR "Urinary Tract Infections/epidemiology" [Majr] OR "Urinary Tract Infections/pathology"[Majr] OR "Urinary Tract Infections/physiopathology" [Majr] OR "Urinary Tract Infections/urine" [Majr]) AND Delirium OR ("Delirium/complications" [Majr] OR "Delirium/diagnosis" [Majr] OR "Delirium/microbiology" [Majr] OR "Delirium/urine" [Majr]) AND Bacteriuria OR ("Bacteriuria/analysis" [Majr] OR "Bacteriuria/diagnosis" [Majr] OR "Bacteriuria/epidemiology" [Majr] OR "Bacteriuria/microbiology" [Majr] OR "Bacteriuria/pathology" [Majr] OR "Bacteriuria/physiopathology" [Majr]) AND Aged)	Age: 65+, Sex: Male & Female, Language: English, Years: 2017 to 2022, Full text articles	37
Science Direct	Urinary tract infection AND delirium	Years: 2017 to 2022	224
Google Scholar	Urinary tract infection induced delirium	Time: 2017 to 2022, Type of article: Any (sorted by relevance)	First 250 articles selected from 12800 results

Results

Study Selection and Quality Assessment

**Figure 1:**
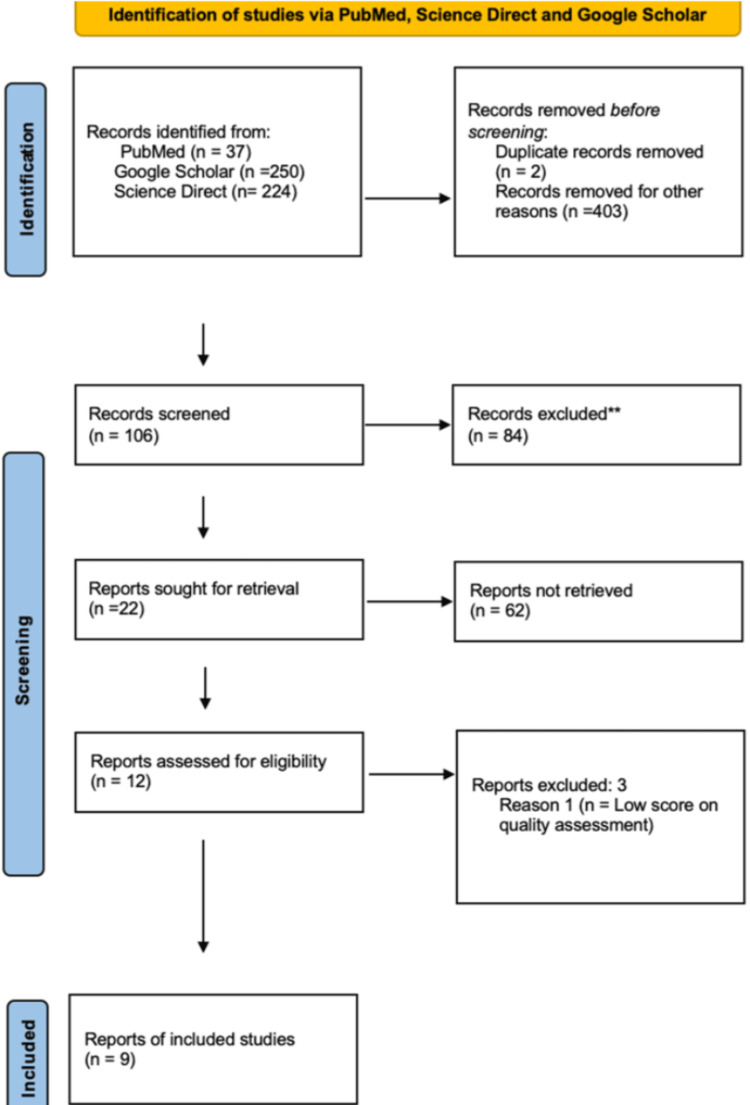
PRISMA 2020 flow diagram for new systematic reviews which included searches of databases and registers only PRISMA: Preferred Reporting Items for Systematic Reviews and Meta-Analyses [10]

A total of 106 articles were identified after applying inclusion/exclusion criteria and removing duplicates in Microsoft Excel. A manual review of those records was done, and 12 studies were selected after determining the most relevant articles. Quality appraisal was done by using the following tools: NOS (The Newcastle-Ottawa Scale), SANRA (Scale for the Assessment of Narrative Review Articles), AMSTAR (Assessment of Multiple Systematic Reviews), and JBI (Joanna Briggs Institute) critical appraisal tool. Every assessment tool had its criteria and scores for quality review. One point was given when a tool scored "1" or "YES," and two points were given when a tool scored "2." Finally, nine studies were selected with scores above 70%, and others were excluded. PRISMA flow diagram for search databases is shown in Figure 1, and tools used for quality assessment of studies are shown in Table 2.

 

**Table 2:** Quality assessment tools and scores of each study selected for this systemic review NOS: The Newcastle-Ottawa Scale; SANRA: Scale for the Assessment of Narrative Review Articles; AMSTAR: Assessment of Multiple Systematic Reviews; JBI: Joanna Briggs Institute

Study name	Quality assessment tool used	Type of study	Score (acceptable >70%)
Wojszel ZB et al. [1]	NOS	Cross-sectional cohort-study	85%
Cortes-Penfield NW et al. [2]	SANRA	Not specified	83%
Rodriguez-Mañas L et al. [3]	SANRA	Not specific	82%
Mayne S et al. [4]	AMSTAR	Systematic review	80%
Magny E et al. [5]	JBI critical appraisal tool	Case series	78%
Wilson JE et al. [6]	SANRA	Not specified	82%
Krinitski D et al. [7]	AMSTAR	Systematic review & meta-analysis	91%
Anand A et al. [8]	SANRA	Not specified	75%
Laguë A et al. [9]	NOS	Observational study	75%

Study Characteristics

The main study characteristics of each selected study are shown in Table 3.

**Table 3:** Main study characteristics of selected studies accepted in this review UTIs: urinary tract infections; DSM-5: Diagnostic and Statistical Manual of Mental Disorders, 5th edition

First author and year	Study type	Disease	Inclusion and exclusion criteria	Sample size/ No. of studies	Outcome and key points
Wojszel ZB et al. 2018 [1]	Cross-sectional cohort study	Urinary tract infection	Women and men >75 years of age	246	UTIs affect 1/5^th^ population of hospitalized elderly patients, and symptoms are often atypical
Cortes-Penfield NW et al. 2017 [2]	Not specified	Urinary tract infection and asymptomatic bacteriuria	-	-	UTIs result in mortality and morbidity in the elderly but inappropriate and early antibiotic usage in treating asymptomatic UTIs can also lead to unfavorable outcomes
Rodriguez-Manas L 2020 [3]	Not specified	Urinary tract infection	Women and men over 65 years of age, asymptomatic bacteriuria, the effect of antibiotic prophylaxis on recurrent UTIs	-	Older adults are more prone to UTIs due to multiple co-morbidities and usually need various medications; therefore, antibiotic use should be minimized, and other treatment options can be explored
Mayne S et al. 2019 [4]	Systematic review	Urinary tract infection	>65 years of age, any care setting, patients with symptoms of UTI and confusion	22	There are unreliable criteria for diagnosis of UTI and confusion; validated criteria for their diagnosis are needed. There is insufficient proof to determine a relationship between afebrile UTI and confusion in the elderly, which requires an in-detail study
Magny E et al. 2018 [5]	Case series	Urinary tract infection and Confusion	Men and women, Diagnosed with Delirium, Patients admitted to Geriatric Acute Care Unit from home, nursing home, or retirement home are included	208	The most frequent precipitating factor of Delirium is an infection, followed by dehydration, electrolyte imbalance, and drugs. A well-directed initial investigation for common precipitating causes of Delirium is needed to improve outcomes
Wilson JE et al. 2020 [6]	Not specified	Delirium	-	-	Several neurobiological processes contribute to the pathogenesis of Delirium. DSM- 5 is the most commonly used tool for the diagnosis of Delirium. The best treatment strategy for Delirium is the identification and treatment of precipitating factors of Delirium
Krinitski D et al. 2021 [7]	Systematic review and meta-analysis	Urinary tract infection, asymptomatic bacteriuria, and Delirium	Aged >65 years with a diagnosis of UTI, Delirium, or asymptomatic bacteriuria and their controls	16,618	A significant association is found between UTI and Delirium in older adults; however, insufficient evidence is found between asymptomatic bacteriuria and Delirium
Anand A et al. 2021 [8]	Not specified	Delirium	-	-	Delirium is associated with a complicated outcome of a disease; therefore, identification of the cause of Delirium and its treatment is needed to limit distress and reduce the length of hospital stay
Lague A et al. 2022 [9]	Observational study	Asymptomatic bacteriuria and Delirium	Physicians who treat older patients with Delirium, speak in French or English and conduct their practice in Canada	297	Treatment of asymptomatic bacteriuria with antibiotics is a regular practice that is non-compliant with the current recommendations. Strict treatment guidelines are needed for treating asymptomatic bacteriuria as the decision to administer antibiotics is often influenced by other physicians and family members

Discussion

This section will include the definition, epidemiology, microbiology, or etiology, risk factors of UTI and delirium in the elderly population, and the diagnostic approach commonly used.

Definitions

This section includes common definitions and explanations of terms used in this systematic review for a better understanding of the readers.

UTI is defined as the presence of localized genitourinary symptoms like increased urine frequency, urgency, painful urination, suprapubic pain, pyuria, and a positive urine culture with an identified urinary pathogen [3,9]. However, there are variations in the criteria of symptoms of UTI across different literatures, worldwide.

Delirium can be explained as a syndrome of severe neuropsychiatric disturbance resulting in a change in awareness, cognition, and fluctuation in attention in a short span due to medical conditions that cannot be described by already existing neurocognitive disorders [6,11].

Asymptomatic bacteriuria is defined as the isolation of bacteria in the urine specimen of a patient who does not have any symptoms of local UTI like dysuria, flank pain, increased frequency, or urgency of urination [12,13].

Epidemiology

UTI contributes to up to 15.5% of hospitalizations and 6.25% of mortalities due to an infectious disease in elderly patients, among which almost one-third of the patients are institutionalized adults [2]. Additionally, UTI can also result in bacteremia in up to 42.6% of male patients with pre-existing diabetes mellitus [14]. Considering the anatomical differences in the urinary tract in men and women, older women are more prone to UTIs than older men; the incidence of UTIs in women is 12.8% compared to men, which is only 7.8% [3].

**Figure 2:**
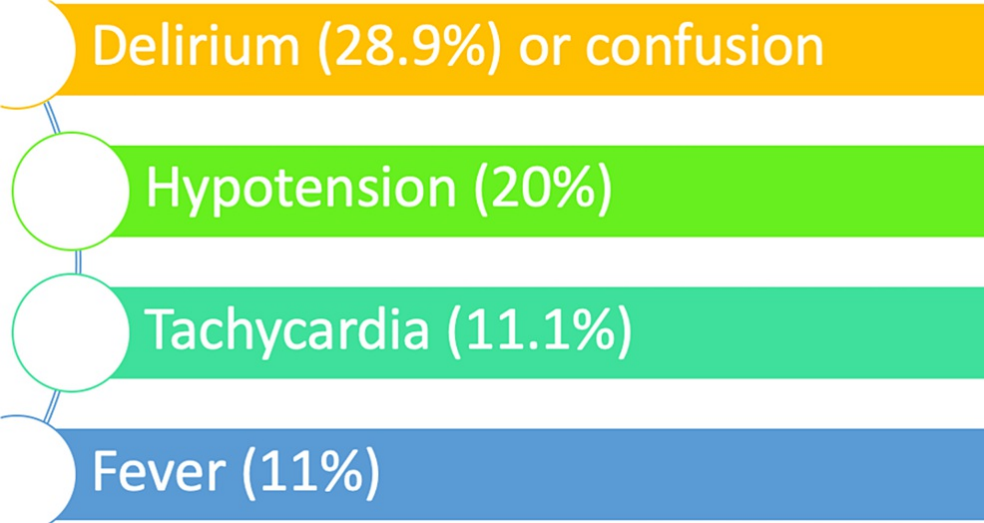
Atypical presentations of UTI in the elderly patients UTI: urinary tract infection (Figure created by the first author)

Based on a prospective cross-sectional cohort study by Wojszel ZB et al. [1], it is concluded that symptoms of UTI are atypical in the elderly population; amongst the people with UTI who participated in the study, only 11% had a fever. Atypical symptoms like delirium were reported in 28.9% of older adults, followed by other symptoms like hypotension (20%) and tachycardia (11.1%) [1], as shown in Figure 2.

There are multiple precipitating factors of delirium, among which infection is the most common precipitating factor seen in up to 49.5% of patients [5,11]. The type of infection that is most common in precipitating delirium is UTI, contributing to up to 15.4% of delirium cases [5].

Microbiology or Aetiology

*Escherichia coli* (*E. coli*) is the predominant pathogen found in 33% of UTI cases, followed by *Klebsiella pneumonia* was found in 22.3% of cases; other pathogens causing UTIs include *Proteus mirabilis,* *Enterococcus faecalis*, methicillin-resistant *Staphylococcus aureus*, *Candida* species, *Enterococcus* species, and *Pseudomonas aeruginosa, *which are commonly seen in patients with catheter-associated UTI [1,3,15,16].

The etiology of UTI-induced delirium may occur due to several possibilities; the first possibility is the inflammatory process involved in the pathophysiology of UTI, which can precipitate delirium; the second possibility is that delirium increases the risk of acquiring UTI as patients may not be capable of maintaining adequate personal hygiene; and the third possibility, changes in physiological systems due to other health conditions that may cause biological dysfunction, which can present as shared risk factors for both delirium and UTI [7]. However, the pathophysiology of delirium is still very speculative [17].

Risk Factors

Risk factors for UTI in the elderly are diverse, including malnutrition, several co-morbidities like diabetes mellitus, urinary incontinence, urinary retention, vaginal atrophy, benign prostate hyperplasia, chronic prostatitis, consumption of multiple drugs for various illnesses, overuse of antibiotics, constipation, cognitive impairment, indwelling urinary catheters, and recurrent UTIs. Amid the numerous risk factors in the elderly, the predictor of the outcome of UTIs is recurrent UTIs and indwelling urinary catheters. In addition, the risk of complications of UTIs like urosepsis dramatically increases with age and is responsible for unfavorable outcomes of infections in the elderly [1-3,9].

A previous history of UTI increases the risk of developing subsequent future UTIs by four to seven folds than in individuals with no past medical history of UTI [3]. The incidence and risk of developing UTIs are higher in women than in men, mainly due to short urethra in women and vaginal atrophy or dryness as a result of a decrease in estrogen [3].

Age is a risk factor for delirium in infectious or inflammatory conditions due to disturbances in optimal oxygenation of the brain, as the increasing age makes the brain more susceptible to the effects of circulating inflammatory particles, which result from inflammatory conditions or infections like UTIs [6]. Prolonged duration and severity of delirium are the factors associated with poor outcomes like short-term mortality, longer duration of hospitalization, and development of dementia [5,6,8]. Once dementia develops, detecting UTI, or UTI-induced delirium becomes difficult [7].

Patients with the presence of bacteria in urine without symptoms of UTI are termed asymptomatic bacteriuria. There is no evidence to link delirium with asymptomatic bacteriuria or vice versa, but the early antibiotic treatment of asymptomatic bacteriuria may further precipitate delirium [7,9]. Many clinicians treat asymptomatic bacteriuria with antibiotics, which increases the risk of antibiotic resistance and the precipitation of delirium [7]. It is reasonable to conclude that bacteriuria in the absence of urinary symptoms does not play any role in the development of delirium [18]. Antibacterial treatment is indicated in the presence of UTI symptoms only and not in asymptomatic bacteriuria [3,19]. Asymptomatic patients should be observed for the development of urinary or systemic symptoms before prescribing antibiotics [19]. A thorough assessment is indicated before giving antibiotics to elderly frail patients [9].

Diagnosis

Signs and symptoms of pre-existing co-morbidities make the diagnosis of UTI difficult as the clinical assessment becomes challenging; for example, conditions like delirium, deafness, and cognitive difficulties (dementia) make communication a complicated obstacle in diagnosing the clinical disease as patients cannot accurately report their symptoms or problems [1,7].

**Figure 3:**
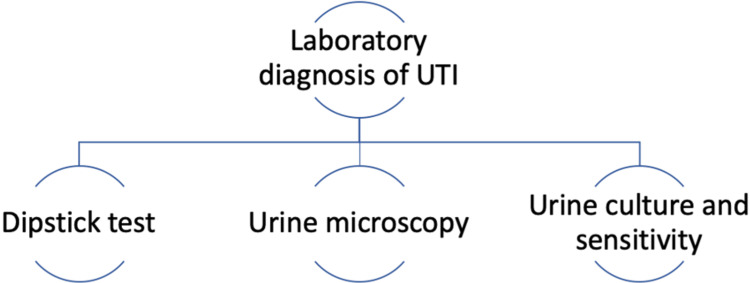
Laboratory diagnosis tests for UTI UTI: urinary tract infection (Figure created by the first author)

Laboratory diagnosis of UTI mainly involves three tests, urine dipstick analysis, urine microscopy, and urine culture and sensitivity [11], as shown in Figure 3.

Urine dipstick allows analysis of leucocyte esterase, which is increased in the presence of urine infection, nitrites, which suggests the presence of bacteria, and red blood cells, which supports the diagnosis of UTI in the presence of positive leukocyte esterase and nitrite [12,19]. In the case of a UTI, urine microscopy shows the presence of more than 10 leucocytes per high-power field, which is termed pyuria, and the presence of bacteria termed bacteriuria [2,19]. Urine culture and sensitivity are considered the gold standard for diagnosis of UTI, the presence of more than 1,00,000 colony-forming bacteria of a single organism in urine is diagnostic of UTI. Urine culture also gives antibacterial sensitivity to the causative organism, thereby helping in the treatment of UTIs. However, this test is expensive, time-consuming, and difficult to obtain without contamination in emergency departments [2,19-21].

Imaging is usually unnecessary in diagnosing uncomplicated UTIs, and it is indicated in patients with UTIs who do not respond to antibiotics and in patients with recurrent episodes of UTIs to identify underlying complications [21,22]. Ultrasound (USG) is the initial imaging technique used in the detection of complicated UTIs [23]; other modalities are computed tomography (CT) scan and MRI (magnetic resonance imaging), which help diagnose complications of UTIs like pyelonephritis, renal or perinephric abscess [24].

Delirium can develop as a result of various physiological and pathological insults; therefore, diagnosis is vital for a better outcome of the disease [6]. The Diagnostic and Statistical Manual of Mental Disorders, 5th edition (DSM-5) helps make a standard diagnosis of delirium. Still, other tools are also available to diagnose delirium depending on different clinical settings [6]. Table 4 shows the DSM-5 criteria for the diagnosis of delirium [8].

**Table 4:** The DSM-5 criteria for diagnosis of delirium DMS-5: Diagnostic and Statistical Manual of Mental Disorders, 5th edition

DSM-5 criteria for diagnosis of delirium
1. Disruption in attention (i.e., reduced ability to focus) and awareness (i.e., reduced orientation to environment)
2. The disturbance develops over a short period, represents a change from baseline attention and awareness, and fluctuates in severity during the day
3. Additional disturbance in cognition (i.e., disorientation of language, perception, memory changes, or visuospatial ability)
4. The disturbances in criteria 1 and 3 are not explained by another pre-existing, evolving, or established neurocognitive disorder and do not take place in the context of a severely reduced level of arousal such as coma
5. There should be indications from the history, physical exam, or laboratory findings that the disturbance is a direct outcome of another medical condition, substance intoxication or withdrawal, exposure to a toxin, or multiple etiologies

**Figure 4:**
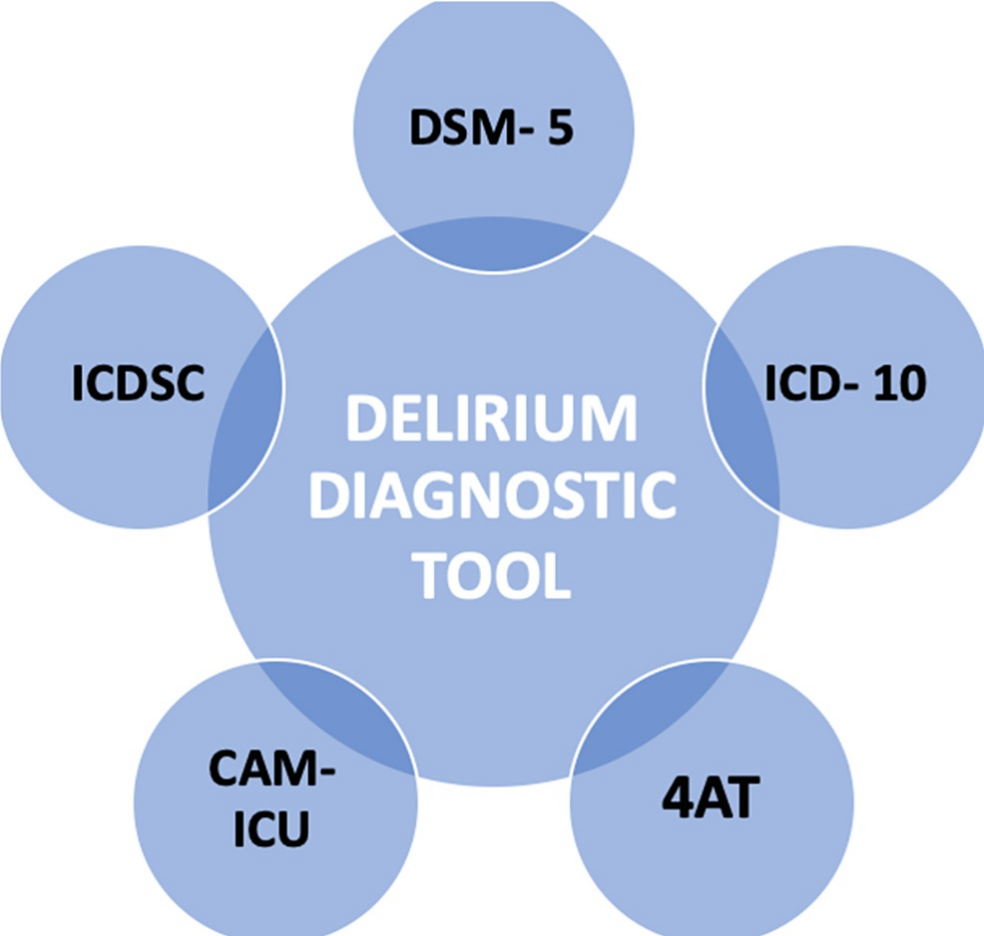
Examples of diagnostic tools for delirium DSM-5: Diagnostic and Statistical Manual of Mental Disorders, 5th edition; ICD-10: The International Classification of Disease, 10th edition; 4AT: 4 'A's Test; CAM-ICU: Confusion Assessment Method for the ICU; ICDSC-Intensive Care Delirium Screening Checklist (Figure created by the first author)

Other tools for diagnosis of delirium shown in Figure 4 include (i) The International Classification of Disease, 10th edition (ICD-10), which requires the presence of symptoms like consciousness and attention, global disturbance of cognition, psychomotor disturbance, sleep-wake cycle disturbance, and emotional disturbances [6]; (ii) 4AT (4 'A's Test) is useful for quick diagnosis, which does not require special training and can be done only in 2 minutes, the four parts of the test include alertness, Abbreviated Mental Test-4 (AMT-4), attention, and presence of acute change or fluctuating course [6,25]; (iii) Intensive Care Delirium Screening Checklist (ICDSC) [6]; and (iv) CAM-ICU (Confusion Assessment Method for the ICU) can be performed for critically ill patients in ICUs [6,26].

Usually, when elderly patients present with symptoms of delirium, the diagnostic approach is toward neurological aetiologies, but data shows that the majority of cases of delirium occur as a result of infection; therefore, the diagnostic approach should be aimed mainly toward searching for infections in the body [5]. However, when a patient presents with delirium, drug intoxication, hypoglycemia, electrolyte imbalances, opioid toxicity, and thyroid dysfunction should also be investigated [8].

Limitations

This study has certain limitations, which should be mentioned to avoid bias. The limitations include variable criteria of symptoms for diagnosis of UTI found across different works of literature, which may have caused overdiagnosis and statistical overestimation of UTI cases. One of the possible explanations is asymptomatic bacteria. In addition, delirium is a common presentation of UTI in the elderly. Still, other undiagnosed underlying causes of delirium in hospitalized patients may make the diagnosis of UTI an incidental finding or association. Furthermore, the findings of this review cannot be applied to all cases of UTI because the clinical presentations vary among the elderly population. Additionally, to maintain the focus of this systematic review on the relationship between UTI and delirium in elderly patients, only the epidemiology, risk factors, etiology, and diagnosis are discussed in detail. However, the treatment and prevention of UTI-induced delirium are not extensively discussed in this article.

## Conclusions

Delirium is a common atypical clinical presentation of UTI in the older population. Delirium can be precipitated by UTI, or UTI can occur due to impaired maintenance of personal hygiene as a result of delirium, leading to unfavorable outcomes. Therefore, there is a valid and complex relationship between them. However, there is no evidence of asymptomatic bacteriuria with delirium. In most cases, UTI-induced delirium is reversible. Various diagnostic tools are available for diagnosing UTIs but the presentations of the infection are often a source of clinical confusion.

This systematic review emphasizes the commonest presentation of UTI in older adults. The aim is to direct clinicians toward the atypical presentation of UTI to minimize the delay in identifying the underlying condition and initiation of treatment. We found that early antibiotic treatment is common in bacteriuria without symptoms of UTI. It is known to precipitate delirium therefore, the initiation of antibiotics should be delayed in asymptomatic patients until a confirmed diagnosis of UTI is established. We also found that there are various criteria of symptoms for the diagnosis of UTI found across different works of literature, which may cause overdiagnosis. Therefore, we recommend that various clinical presentations of UTI like delirium, tachycardia, and hypotension should be identified and studied to prevent delay in treatment. Furthermore, there are multiple explanations available for UTI-induced delirium, a well-designed study will help in clarifying the exact pathophysiology of UTI-induced delirium.
